# Use of Magnetic Resonance Imaging in Evaluating Fetal Brain and Abdomen Malformations during Pregnancy

**DOI:** 10.3390/medicina55020055

**Published:** 2019-02-17

**Authors:** Nomeda Rima Valevičienė, Guoda Varytė, Jolita Zakarevičienė, Eglė Kontrimavičiūtė, Diana Ramašauskaitė, Dileta Rutkauskaitė-Valančienė

**Affiliations:** 1 Department of Radiology, Nuclear Medicine and Medical Physics, Faculty of Medicine, Institute of Biomedical Sciences, Vilnius University, M. K. Čiurlionio g. 21, 03101 Vilnius, Lithuania; nomeda.valeviciene@santa.lt (N.R.V.); dileta.rutkauskaite@santa.lt (D.R.-V.); 2 Faculty of Medicine, Vilnius University, M. K. Čiurlionio g. 21, 03101 Vilnius, Lithuania; 3 Faculty of Medicine, Institute of Clinical Medicine, Vilnius University Clinic of Obstetrics and Gynaecology, Vilnius University Hospital Santaros Clinics, Santariškių g. 2, 08661 Vilnius, Lithuania; jolita.zakareviciene@santa.lt (J.Z.); diana.ramasauskaite@santa.lt (D.R.); 4 Faculty of Medicine, Institute of Clinical Medicine, Vilnius University Centre of Anaesthesiology, Intensive Care and Pain Treatment, Vilnius University Hospital Santaros Clinics, Santariškių g. 2, 08661 Vilnius, Lithuania; egle.kontrimaviciute@santa.lt

**Keywords:** fetal MRI, pregnancy, gadolinium

## Abstract

Magnetic resonance imaging (MRI) is used as a clarifying technique after a high-resolution ultrasound examination during pregnancy. Combining ultrasound with MRI, additional diagnostic information is obtained or ultrasound diagnosis is frequently corrected. High spatial resolution provides accurate radiological imaging of internal organs and widens possibilities for detecting perinatal development disorders. The safety of MRI and the use of intravenous contrast agent gadolinium are discussed in this article. There is no currently available evidence that MRI is harmful to the fetus, although not enough research has been carried out to prove enduring safety. MRI should be performed when the benefit outweighs the potential side effects. The narrative review includes several clinical cases of fetal MRI performed in Vilnius University Hospital Santaros Clinics.

## 1. Introduction

Although ultrasound remains a major and preferred radiological study during pregnancy, due to its wide availability, low cost and safety, the benefits and possibilities of using magnetic resonance imaging (MRI) are rapidly developing. During pregnancy, a high-resolution ultrasound examination is usually followed by magnetic resonance imaging to clarify the results. The quality of MRI is not affected by factors such as maternal fatty layer, fetal position, overlap of bone structures and oligohydramnios. The optimal time to perform MRI depends on which pathology is suspected by ultrasound. Fetal organs up to 18 weeks are underdeveloped, fetal movements are increased, and some pathologies might not have been developed yet (i.e., cortical dysplasia) [1]. Although MRI can be performed at any time during pregnancy, it is usually performed at 20 weeks pregnant and onwards, when the fetal organs are already of sufficient size to be visualised. A team of specialists is often required with experience in obstetrics, gynecology and fetal magnetic resonance imaging [2]. After MRI is performed, the fetus anatomy is evaluated in detail. During this procedure, the examiner evaluates the volume of amniotic fluid, the placenta’s position, its size and signal intensity in the uterus, the length and anatomy of the umbilical cord, the maternal uterus and the adnexa. The birth canal can also be assessed [3]. In this paper we discuss the two cases of magnetic resonance examination performed for fetal brain and abdomen malformations.

## 2. Fetal MRI Sequences

The adoption of ultrafast MRI sequences has led to an extreme improvement of fetal MRI by diminishing artifacts caused by an excessive fetal motion and the necessity to use sedation during this examination. Turbo spin echo is a standard sequence for fetal MRI examination using single time repetition (TR single shot) also known as single-shot fast spin echo (SSFSE). This sequence is based on a single slice acquisition in a very short time repetition (TR) (<3 ms) so the artifacts can be efficiently reduced. Two types of T1-w sequences are used: gradient echo (GE), with short TR and time echo (TE), and fast spin echo (FSE-T1). FSE-T1 grants improved spatial resolution but needs about 20 s of apnea, while GE requires 14–15 s of breath-holding but provides us with reduced spatial resolution. In contrast to T2-weighted SSFSE, slices in the gradient-echo sequence are acquired simultaneously meaning that even a slight fetal movement reflects in all slices as a motion artifact. T1-weighted sequences provide less information compared to T2-weighted SSFSE sequences, however, in T1 sequences, the organs such as pituitary gland, cortex and myelinated fibers are represented by a hyperintensive signal which allows the detection of hemorrhage or fat lesions. Fetal brain contains free fluid that increases the contrast between the cortex, basal ganglia and white matter in T1-weighted sequences with fat suppression [4,5].

## 3. Evaluating the Fetal Brain

Fetal magnetic resonance is a particularly informative technique to evaluate sonographically suspected abnormalities of the central nervous system. The most frequent fetal MRI indications are ventriculomegaly, agenesis of corpus callosum and the posterior fossa malformations.

Ventriculomegaly is a brain condition when the diameter of the atrium of the lateral ventricle is equal to or greater than 10 mm. Ventriculomegaly can be split into three groups: mild (10–12 mm), moderate (12–15 mm) and severe (>16 mm). Etiology is determined by obstructive, destructive and developmental factors meaning ventriculomegaly can be associated with additional CNS abnormalities or isolated. The differentiation between isolated ventriculomegaly and secondary to another type of pathology is very important as it determines the clinical prognosis. Mild ventricle dilatation and no additional CNS anomalies are associated with better neurodevelopmental outcomes. In terms of the benefits of ultrasound scan and magnetic resonance imaging, MRI is more beneficial due to its spatial resolution and detailed visualisation of the evolving parts of the brain, as well as detection of malformations in addition to ventricle dilatation. Apart from ventriculomegaly, the findings frequently involve cortical and cerebellar malformations, periventricular heterotopia, hemimegaloencephaly and periventricular and germinal matrix hemorrhage [4,5,6]. P.D. Griffits et al. conducted a prospective study of 147 cases of fetuses with isolated ventriculomegaly examined by ultrasound and MRI; 17% of the fetuses were diagnosed with additional pathologies of the central nervous system, in most cases, corpus callosum agenesis [7].

Corpus callosum is a hypointense structure that can be identified from the 20th week of gestation in T2-weighted sagittal images. It can be divided into four parts that correspond to the development sequences: genu, splenium, truncus and rostrum [3]. Agenesis of corpus callosum might be partial or complete and each of them can be isolated or be in a complex with other abnormalities. Fetal MRI is a useful tool for a detailed corpus callosum visualisation. It enables distinguishing between the isolated and secondary agenesis of corpus callosum and detects additional pathology unnoticed by ultrasound. Hetts et al. [8] retrospectively evaluated commissural abnormalities and the presence of coexisting structural anomalies. The isolated agenesis of corpus callosum in the examined patients was infrequent; grey matter heterotopia and malformations of cortical development (polymicrogyria, lissencephaly, pachygyria, shizencephaly) were diagnosed in more than half of the patients.

Ultrasound visualisation of fetal brain structures, including posterior fossa, becomes very complex in the last trimester of pregnancy due to ossification of the skull. Malformations detected after MRI examination include Dandy–Walker and Chiari II malformation, cerebellar hypoplasia, dysplasia and hemorrhage. Fetal MRI is a necessary tool for proper visualisation of the brainstem, tentorium cerebelli position, cerebellar hemispheres, vermis, cisterna magna and the size of the fourth ventricle. When vermis malformation is examined by ultrasound, fetal MRI is mandatory to evaluate its diameter, anatomical structure and its relations between the fourth ventricle and the retrocerebellar cyst, or to confirm its nonappearance [4,6].

**Figure 1:**
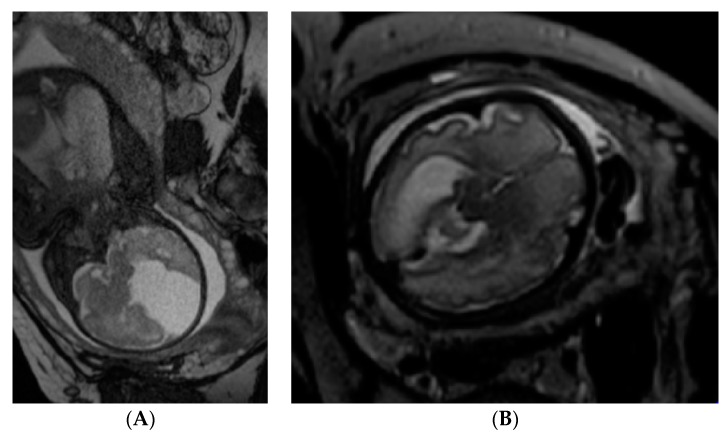
(**A**,**B**) Coronal and axial MRI scans of the fetuses brain. Left posterior and temporal horn was dilated—18 mm. From cisterna ambient interhemispheric fissure O-P-F and liquor collections O-P-F 6.7 × 3.3 cm with septum. Signs of corpus callosum agenesis.

The fetal MRI (Figure 1A,B) was performed in Vilnius University Hospital Santaros Clinics on a 25-year-old, 31-week + 4-day-pregnant patient whose high-resolution ultrasound scan had indicated the left side brain ventriculomegaly, choroid plexus cysts on the right side and the deformed midline of the brain. MRI was performed without using an intravenous contrast agent and revealed a significant right-side brain hypoplasia with a gigantic interhemispheric cyst, ventriculomegaly on the left, signs of corpus callosum agenesis.

## 4. Evaluating the Fetal Abdomen

Fetal MRI is performed to evaluate the gastrointestinal tract (GI) in cases where anomaly is suspected by prenatal sonography. Most common indications to fetal GI MRI involve the obstruction of esophagus and small and large bowel, and malrotation and perforation of fetal GI. MRI is significant in detecting the abdominal wall defects such as diaphragmatic hernia, gastroschisis and omphalocele [9].

Gestational age is important in visualising the gastrointestinal track. Even though the fetus starts swallowing from 9 to 10 gestational weeks, a sufficient amount of amniotic fluid does not enter the gastrointestinal tract of the fetus until 25 weeks of gestation and serves as a native contrast material [10]. The fetal gastrointestinal tract is filled up with meconium and amniotic fluid. The upper part of the gastrointestinal tract (esophagus, stomach, duodenum) is normally full of swallowed T2-hyperintense amniotic fluid which serves as an intrinsic contrast media in diagnosing pathology in the oral cavity, pharynx and larynx. T2-hypointense and T1-hyperintense meconium fill distal ileum and colon parts. A high concentration of protein and paramagnetic elements, such as iron, copper and magnesium, determines the meconium signal intensity in the T1-weighted sequence. The signal intensity of the amniotic fluid and meconium mixture serves in identifying different parts of the gastrointestinal tract. The comparison of the upper and lower parts of the gastrointestinal tract shows that in the lower gastrointestinal part, T2-hyperintensity decreases while T1-hyperintensity increases. As a result of this, T2-hyperintense small bowel loops in healthy fetuses should be observed in the upper left quadrant while T2-hypointense loops in the right lower quadrant of the abdomen [9,11].

Atresia, obstruction and stenosis are commonly found in the small intestines, consequently fetal MRI serves in determining the sites of obstruction. T2-hyperintensity and T1-hypointensity indicate the accumulation of amniotic fluid that is common to the upper gastrointestinal tract atresia. A sign of lower atresia or obstruction is a high signal on T1-weighted and low signal on T2-weighted fetal MRI due to collection of meconium [12].

Fetal MRI plays the main role in identifying congenital diaphragmatic hernia (CDH) and evaluating its position with the other structures. The defects of the diaphragm are more likely to be intrapleural and appear on the left side. The left-sided herniation includes omental fat, intestines, left liver lobe and stomach, whereas herniation of the right hepatic lobe is more common to the right-sided CDH. Almost all CDHs are intrapleural, causing lung hypoplasia due to cardiomediastinal displacement. The organs full of amniotic fluid, such as the stomach and small bowel, are visible at SSFSE T2-weighted sequence because of their hyperintense fluid signal. The small bowel loops and the heart are hypointense structures on T2-weighted images. This feature helps to differentiate between the liver that is normally hyperintense in the T1 FFE (fast field-echo) images. A balanced SSFP (bSSFP) image allows the examiner to identify vessels within liver parenchyma as the circulation of blood is hyperintense on bSSFP sequence. In cases of liver herniation through the diaphragmatic defect, hyperintense vessels in hepatic parenchyma can be observed extending above the diaphragm level [13,14].

**Figure 2:**
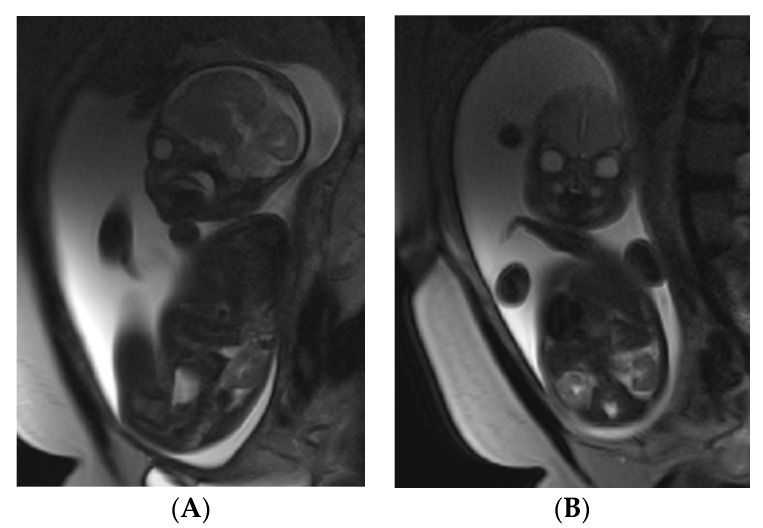
(**A**,**B**) Sagittal and coronal MRI scan of the fetus. Herniation in the left side of the diaphragm, the stomach, bowel loops and the left liver lobe in the chest cavity.

A fetal MRI (Figure 2A,B) without intravenous contrast agents was performed in Vilnius University Hospital Santaros Clinics on a 27-year-old female patient for a suspected left-side diaphragmatic hernia. The stomach, bowel loops and left liver lobe in the chest cavity were observed. The right lung was compressed, the left lung was not visible.

## 5. Safety

At the moment there is no proof that MRI is unsafe for the fetus, however, not enough research has been carried out to prove enduring safety. Suggestions are given to carry out MRI after 18 weeks of pregnancy due to unidentified biological outcomes of high magnetic fields during the period of organogenesis [1]. The biological cell response to MRI involves induction of local electric fields and the heating of tissues caused by radiofrequency radiation [15]. Heating can have destructive consequences in the organ developmental period [16]. Hand et al. [17] have revealed that the highest SAR (specific absorption rate) appears in maternal tissues in either 1.5 T or 3 T magnetic fields, while the fetal SAR varies from 40% to 70% of the highest maternal SAR.

There are uncertainties about the fetal MRI, its impact on the fetal growth restrictions in the uterus, premature birth, and the damage to the inner ear due to high acoustic disturbances during the procedure. There was a recent study performed which assessed the fetal development and postpartum inner ear function after exposure to MRI at 1.5 T during pregnancy. The results revealed no negative outcomes after exposure to 1.5 T MRI procedure, meaning that the percentage of the inner ear dysfunction or deafness was found to be zero, nor was there a significant difference in the newborns’ weight [18]. Another similar study was performed by Joel G. Ray et al. [19]. The study was designed to assess the long-term safety after performing MRI during the first trimester of pregnancy. The researchers estimated the risk of a stillbirth or newborn death within 28 days after delivery and any inborn malformation, abnormal growth and hearing or vision disorders until 4 years old. The results of the study have proven that the risk of adverse effects on the fetus or preschool children has not increased after exposure to MRI during the first trimester of pregnancy. Marine Bouyssi-Kobar et al. [20] performed the prospective study to evaluate the functional outcomes in minors who were examined by 1.5 T fetal MRI in the second or third trimester of pregnancy. The authors were not able to establish a link between the inner ear dysfunction in preschool children and the fetal exposure to magnetic fields in the second and third trimester of pregnancy.

The use of gadolinium for pregnant women is highly controversial. Gadolinium is a pregnancy class C drug, meaning that its safe administration to humans has not been confirmed [2,16]. Despite the lack of adequate evidence of gadolinium side effects, the consensus among radiologists is to avoid this contrast material for precautionary reasons. Gadolinium agents injected intravenously cross the placenta barrier and get into the fetal circulation. Gadolinium is nontoxic in the form of chelate and it remains for a long time until it is absorbed and eliminated. Gadolinium is secreted into the amniotic fluid from the fetal bladder. The longer gadolinium chelate form stays in this space, the greater is its risk to dissociate from its chelate molecule and become very toxic. In pregnant patients requiring MRI, the procedure is most commonly performed without gadolinium-enhanced images, taking into account the concern for potential side effects to the fetus [21,22].

In order to provide the data about the contrast material distribution in the mother and fetus, studies have been performed on animals of lower species. Karen Y. Oh et al. conducted a study aimed to estimate gadolinium chelate in Gravid Japanese macaques’ fetal tissues and amniotic fluid after 19–45 hours after intravenous administration of gadosteridol. As a result, the contrast material in the fetoplacental circulation was minimal compared to the injected maternal dose [23]. A few years later, Joao Prola-Netto performed an experimental study with juvenile nonhuman primates in order to determine if the contrast material remained in the juvenile nonhuman primate after giving a pregnant mother an intravenous injection. The presence of the contrast material in amniotic fluid confirmed that, after maternal exposure to gadosteridol, it transits through the placental barrier. The examination of the macaque tissue after birth has revealed the presence of very small gadolinium doses, which proves that very low levels of contrast material remain after birth [24].

## 6. Conclusions

Fetal MRI is a rapidly evolving technique in perinatology due to its accuracy and excellent quality of images. In many cases, the combination of fetal MRI with high-quality ultrasound facilitates additional diagnostic information which widens the possibilities for detecting early perinatal development disorders. Fetal MRI does not use ionizing radiation and is assumed to be safe, however, there is not enough evidence to prove its long-term safety.
